# Terrible Triad Injury of the Elbow Associated With an Olecranon Fracture: A Case Report With Three-Year Follow-Up

**DOI:** 10.7759/cureus.103734

**Published:** 2026-02-16

**Authors:** Nikolaos P Sachinis, Eleni Karagergou, Menelaos Mountzouris, Alexandros Givissis, Givissis K Panagiotis

**Affiliations:** 1 First Orthopaedic Department, Aristotle University of Thessaloniki, General Hospital "Georgios Papanikolaou", Thessaloniki, GRC; 2 Department of Orthopaedics, School of Medicine, European University Cyprus, Nicosia, CYP; 3 First Orthopaedic Department, Aristotle University of Thessaloniki, Faculty of Medicine, General Hospital "Georgios Papanikolaou", Thessaloniki, GRC

**Keywords:** elbow, elbow fracture, olecranon, orthopaedic, terrible tetrad, terrible triad, terrible triad plus olecranon, trauma

## Abstract

Terrible triad injuries of the elbow consist of elbow dislocation associated with fractures of the radial head and coronoid process and are characterized by significant instability. Variants that include an associated olecranon fracture are rare, and data regarding long-term functional outcomes and patient-reported outcome measures remain limited.

We report the case of a 74-year-old female patient who sustained a terrible triad injury of the elbow associated with an ipsilateral olecranon fracture following a low-energy fall. This injury pattern has occasionally been referred to as a “terrible tetrad”; however, this term is not uniformly adopted in the literature. Unlike trans-olecranon fracture-dislocations, the present injury involved disruption of the ligamentous stabilizers, necessitating soft-tissue reconstruction in addition to osseous fixation. Surgical treatment was performed through a single posterior approach and included olecranon fixation, radial head arthroplasty, coronoid fixation, and repair with augmentation of the lateral collateral ligament complex. At three-year follow-up, the elbow was stable, with a flexion-extension arc from 25° to 115° and full forearm rotation. The Mayo Elbow Performance Score was 85, and the QuickDASH score was 4.5. Radiographs demonstrated fracture union, a well-positioned radial head prosthesis, heterotopic ossification, and early post-traumatic degenerative changes.

This case highlights that comprehensive reconstruction addressing both osseous and ligamentous instability can result in acceptable mid-term functional outcomes in this uncommon injury pattern.

## Introduction

The terrible triad injury of the elbow is defined as the combination of elbow dislocation, radial head fracture, and coronoid process fracture and represents a complex fracture-dislocation associated with global elbow instability [1]. Biomechanical studies have demonstrated that this injury results from a sequential failure of the lateral and medial stabilizing structures, leading to instability that cannot be addressed by isolated osseous fixation alone [1]. This pattern is distinct from trans-olecranon fracture-dislocations, in which the ulnohumeral articulation remains congruent if the proximal ulna is fixed and instability is primarily osseous rather than ligamentous [2].

However, the available literature on terrible triad injuries associated with olecranon fractures remains limited. Terrible triad injuries may present with associated fractures, including olecranon fractures, but published evidence for this variant remains limited and long-term outcome reporting is scarce [3]. Published reports consist predominantly of single cases or small series, are characterized by short follow-up periods, and frequently lack validated patient-reported outcome measures, restricting interpretation of mid- to long-term functional results [4,5].

The purpose of this case report is to present the three-year clinical and functional outcomes of a patient with a terrible triad injury associated with an ipsilateral olecranon fracture, treated surgically with radial head arthroplasty, ligament repair and augmentation, and olecranon fixation, with outcome assessment using range-of-motion measurements and validated functional scores.

## Case presentation

A 74-year-old female patient presented to the emergency department after a low-energy fall from standing height, reporting acute pain, swelling, and functional impairment of the left elbow. Clinical examination demonstrated marked swelling, deformity, and severe limitation of motion. Neurovascular examination of the affected limb was normal.

Plain radiographs of the elbow demonstrated a posterior elbow dislocation associated with fractures of the radial head and coronoid process, consistent with a terrible triad injury. An associated fracture of the olecranon was also identified. Closed reduction of the elbow dislocation was performed in the emergency department, followed by immobilization. Post-reduction neurovascular status remained intact.

**Figure 1:**
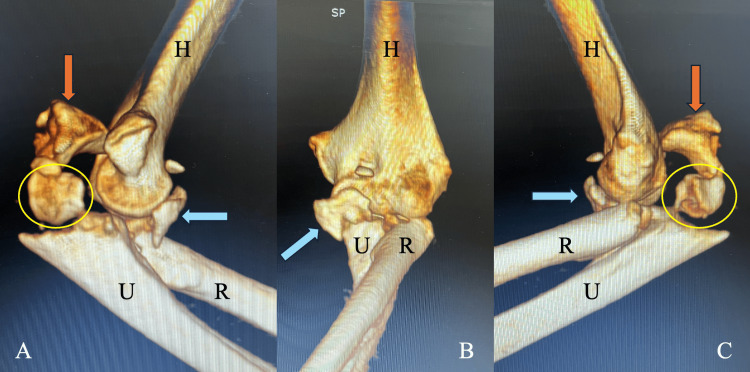
Three-dimensional computed tomography reconstructions of the left elbow demonstrating a terrible triad injury associated with an olecranon fracture. (A) Medial view showing the radial head fracture (yellow circle), coronoid fracture (blue arrow), and olecranon fracture (orange arrow). (B) Anterior view highlighting the coronoid fracture (blue arrow). (C) Lateral view demonstrating the radial head fracture (yellow circle), coronoid fracture (blue arrow) and posterior displacement of the olecranon fragment (orange arrow). H: humerus; U: ulna; R: radius.

Computed tomography of the elbow was subsequently performed to fully delineate the osseous injury pattern and confirmed the presence of a radial head fracture, coronoid process fracture, and olecranon fracture (Figure 1).

Based on the imaging findings, surgical treatment was indicated due to the complexity of the injury and the risk of residual instability. Regarding bone density status, the patient had recently undergone a dual-energy X-ray absorptiometry scan of her right hip, showing normal density (T score: 0.6; Z score: 1.2).

Surgical treatment was performed through a single posterior approach incorporating a posterolateral interval to the elbow. The step list of this procedure involved initial treatment of the olecranon, coronoid, then the radial head and finally the lateral collateral complex. The olecranon fracture was addressed with internal fixation using a tension band technique. The coronoid fracture was stabilized using two partially threaded screws. The radial head was deemed non-reconstructible and was treated with radial head arthroplasty. The lateral collateral ligament complex was repaired and augmented using a non-absorbable suture tape and anchors construct to restore lateral elbow stability and protect the ligament repair during early mobilization. Intraoperative assessment following fixation and soft-tissue reconstruction demonstrated a stable elbow through a functional range of motion.

Post-operatively, the elbow was immobilized for seven days in a cast and then mobilized according to a structured rehabilitation protocol emphasizing early controlled passive range of motion for the first six weeks and active after that period. No perioperative complications were recorded.

At one-year follow-up, the patient demonstrated satisfactory elbow stability and functional recovery, with a flexion-extension arc of 10° to 105°. The Mayo Elbow Performance Score was 100 points, and the QuickDASH score was 2.3 [6,7]. These scores are free to use.

**Figure 2:**
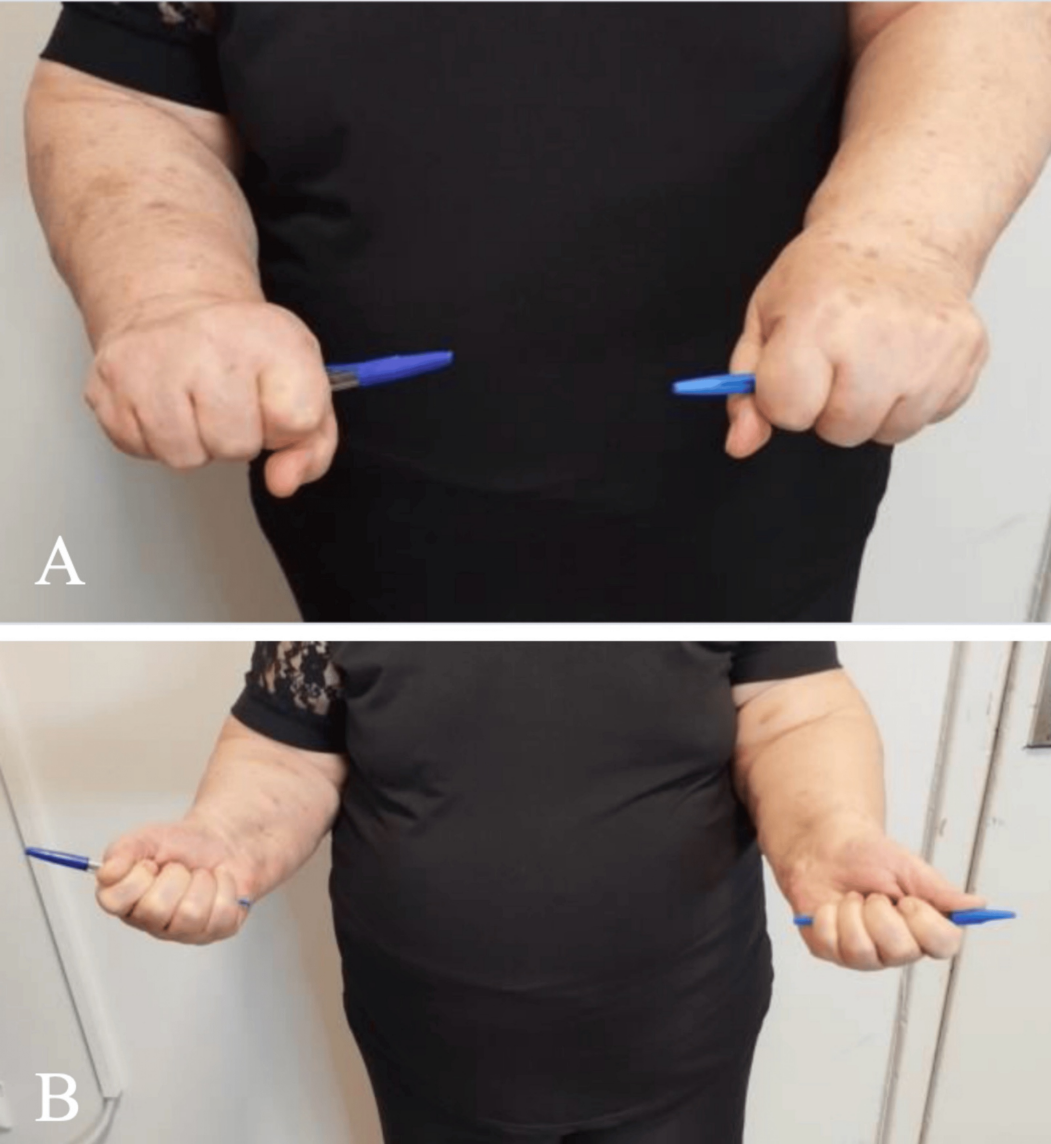
Clinical photographs demonstrating forearm rotation at the final follow-up. (A) Full pronation. (B) Full supination.

**Figure 3:**
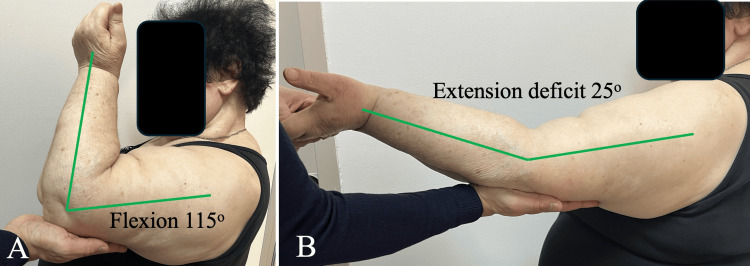
Clinical photographs demonstrating elbow range of motion at the final follow-up. (A) Active elbow flexion to approximately 115°. (B) Elbow extension with an extension deficit of approximately 25°.

At the final follow-up, three years after surgery, the patient reported occasional mild pain during daily activities but no episodes of instability. Elbow range of motion, measured with a standard goniometer, demonstrated full pronation and supination, and a mild loss of extension measuring approximately 25° and flexion improved to approximately 115° (Figures 2, 3).

Measurement accuracy was partially limited by the patient’s soft tissue envelope. The Mayo Elbow Performance Score at the final follow-up was 85 points, reflecting a functional but limited flexion-extension arc, possibly due to progressive limitation in flexion-extension and the development of post-traumatic degenerative changes, despite maintained joint stability. The QuickDASH score was 4.5, indicating minimal residual disability.

**Figure 4:**
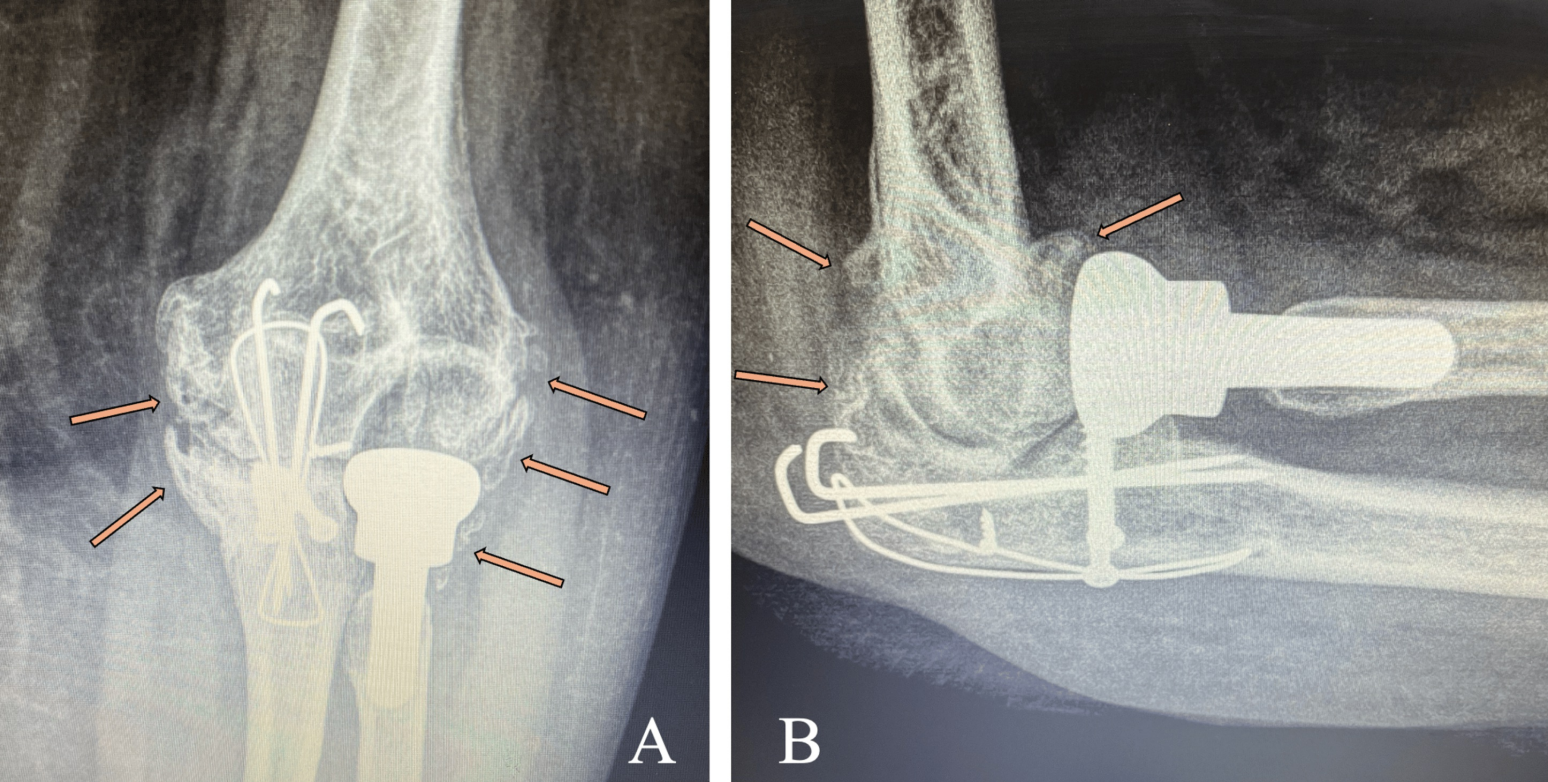
Anteroposterior (A) and lateral (B) radiographs of the left elbow at the final follow-up, demonstrating a well-positioned radial head prosthesis, union of the olecranon fracture, heterotopic ossification (orange arrows), and early-stage post-traumatic degenerative changes.

Radiographic evaluation at the final follow-up demonstrated a well-positioned radial head prosthesis with mild stress shielding, without clinical or radiographic evidence of loosening. The olecranon fracture had united. Heterotopic ossification and early post-traumatic degenerative changes were noted around the elbow joint (Figure 4).

## Discussion

Terrible triad injuries of the elbow are complex fracture-dislocations characterized by radial head and coronoid fractures associated with disruption of the ligamentous stabilizers, resulting in predictable global instability if not adequately addressed [1,8]. Surgical management is therefore directed toward restoration of stability rather than anatomic reconstruction alone, allowing early mobilization and functional recovery [8-10]. In the present case, comprehensive reconstruction addressing both osseous and ligamentous components resulted in a stable and functional elbow at three years of follow-up.

Variants of the terrible triad injury that include an associated olecranon fracture have been infrequently reported. In a systematic review, Medina et al. identified olecranon fracture as the most common additional osseous injury among terrible triad variants; however, available evidence was limited to isolated case reports and small series with heterogeneous treatment strategies and short follow-up [3]. Individual reports describing this injury pattern, sometimes referred to as a “terrible tetrad,” have emphasized the severity of the combined osseous and soft-tissue injury and the need for comprehensive reconstruction to restore elbow stability [4,5]. In this context, radial head arthroplasty has been advocated for unreconstructible fractures to achieve predictable stability, while ligament repair remains essential given the ligamentous insufficiency inherent to this injury pattern [2,8,11].

At three years post-operatively, the patient demonstrated a functional flexion-extension arc with full forearm rotation and minimal residual disability, as reflected by a Mayo Elbow Performance Score of 85 and a QuickDASH score of 4.5. These findings are comparable to functional outcomes reported following surgical treatment of standard terrible triad injuries, despite the additional complexity introduced by the olecranon fracture [8,9]. The use of validated patient-reported outcome measures such as the QuickDASH provides a meaningful assessment of functional recovery and complements objective clinical evaluation [7,12].

Radiographic evaluation demonstrated heterotopic ossification and early post-traumatic degenerative changes. Heterotopic ossification has been reported in a substantial proportion of surgically treated elbow fracture-dislocations, particularly in complex injuries involving combined osseous and ligamentous trauma [13]. Post-traumatic osteoarthritis is also a recognized consequence following terrible triad reconstruction and has been associated with a reduced range of motion and lower functional scores, even in clinically stable elbows [14]. The mild intermittent pain and motion limitation observed in the present case are consistent with these findings.

Although the three-year follow-up of this case report provides meaningful mid-term data, longer observation would be required to assess the progression of degenerative changes and long-term implant survivorship. In addition, range-of-motion measurements were partially influenced by the patient’s soft-tissue envelope, despite the use of a standard goniometer.

## Conclusions

Terrible triad injuries associated with an ipsilateral olecranon fracture represent a rare and particularly complex variant of elbow fracture-dislocation. This case demonstrates that comprehensive reconstruction addressing both osseous and ligamentous instability can result in a stable elbow with acceptable function at mid-term follow-up. Further reports with longer follow-up and validated patient-reported outcome measures are needed to better define expected outcomes for this uncommon injury pattern.
